# Resident work hours: why keeping the status quo may not be such a bad thing

**Published:** 2013-09-30

**Authors:** Roshan Razik, Marat Slessarev

**Affiliations:** University of Toronto

## Abstract

Resident duty hours have become an increasingly debated topic in post-graduate medical education. Work-hour restrictions have been implemented for first-year residents in the US and more recently for all residents in Quebec. Current and future work-hour rules affect a variety of stakeholders: government, hospitals, residency training programs, patients, and most of all residents. In this article, we hope to examine the issue from a Canadian perspective and delineate some of the reasons why changing the current call structure may have potentially deleterious effects to all those concerned.

## Introduction

On June 7^th^ 2011, a Quebec court ruled that the current model of on-call shifts for resident physicians is in violation of the Canadian Charter of Human Rights.^1^ This resulted in the Fédération Médecins Résidents Québec (FMRQ) setting restrictions on working hours from 24+2 on-call models (24 consecutive hours on call with two hours for handover) to a 16 or even 12-hour call shift, setting off a wave of similar initiatives in other Canadian provinces. Improvement in patient safety and resident well-being are quoted as the main drivers for this historic change, supported by evidence primarily originating from work done in US hospitals. Although these initiatives are exciting, like any change, they may lead to a number of unintended consequences. We aim to highlight some of these issues in this paper with the intention to warn residents and the public to carefully consider the potential drawbacks associated with the proposed change prior to its implementation.

### The doctor-patient relationship

William Osler’s vision for residency training was to have newly graduated doctors work alongside established clinicians, as they learned to practice independently, gradually taking on more responsibility.^2^–^4^ Osler was aware of the importance of developing professional responsibility for patients - the sense of ownership of specific patients that is central to the practice of inpatient medicine and surgery. Switching to shorter workdays may cause residents to lose this fundamental part of their educational experience,^5^,^6^ possibly impeding residents from recognizing complications resulting from their treatment plans and preventing them from learning from their own mistakes.

Although some specialties such as radiology, anesthesia and emergency medicine lend themselves to a shift-based work schedule, they are not admitting specialties and do not carry the label of the physicians most responsible for inpatients. By their very nature, such specialties tend to deal with patients as itemized cases where continuity of care is not as crucial. After a case is completed (e.g., a CT scan dictated or an epidural administered), there is often no ongoing duty to oversee primary responsibility for that patient. Although a fiduciary relationship still exists, the case is often effectively closed, and in most instances, will not be revisited again either during that shift or any time in the future. In such specialties, implementing work-hour restrictions would not lead to disruption in an integral part of the doctor-patient relationship. On the other hand, establishing patient rapport and continuity of care is central to the practice of inpatient medicine and surgery and this skill cannot be acquired in the classroom. Imposing work-hour restriction risks losing this component from resident education, and may lead to dissatisfaction among patients and other members of the healthcare team.^7^

For example, in a randomized trial by Desai and colleagues, implementation of a night-float system had to be terminated early due to increasing concern from nurses about the quality of care provided when the system was introduced.^7^ Furthermore, the ability to perform effectively while managing fatigue may be an essential learned skill that needs to be acquired by trainees during residency in order to work in specialties that require provision of 24-hour emergency and inpatient coverage, given the realities of a healthcare system that is under strain.^8^ Although system-level changes in healthcare to ensure adequate doctor-patient ratios and physician workload should remain a priority, addressing physician shortages, especially in rural areas of Canada, at present requires that physicians work longer hours.^9^,^10^

The effects of time restrictions on residents’ preparedness to practice independently are unknown. Much like army recruits who bypass boot-camp, they may simply be unprepared to face the rigours and demands of an independent practice upon graduation, especially in rural and underserviced areas. In fact, in a survey of 6,202 US interns and residents, more than half (52 %) were of the opinion that preparation for more senior roles was worse with the implementation of the 16-hour limit for interns.^11^ This may be due in part to a perception among 66% of respondents that new rules have simply shifted the work from interns to senior residents, and 41% of respondents seeing worsening in quality of education.^11^ Similarly, Fletcher et al. showed that residents who were forced to comply with new duty hour limits were readily willing to “break the rules”: over two thirds of trainees in this study wanted to stay past the prescribed work-hour limits and often cited the need to provide continuity of care to an acutely ill patient or to allow for ‘humanistic attention’ to a particular patient or family.^12^

### Handovers and night-float systems

Shorter shifts mean more handovers and therefore more room for error in patient care.^11^,^13^,^14^ Many studies have stressed the importance of comprehensive patient handover and have shown adverse outcomes with poor communication at various points in the transition of patient care.^13^,^15^,^16^ The accuracy of patient handover in predicting adverse events has also been questioned.^17^,^18^ Despite recognizing the importance of good handover, effective implementation in the real-world setting has been difficult and only in the last few years have recommendations been made to formalize the process and provide specialty-specific guidelines.^16^

With proposed duty-hour restrictions, many new call models have introduced night-float residents who work a series of back-to-back night shifts. Although they work shorter hours, a well-rested resident *does not* automatically equate with better care. Frequently, these residents may be unfamiliar with the day team’s overall plan for a group of patients whose illnesses evolve over a longer time frame than a 12-hour shift would allow. This could lead to confusion, mixed messages and adverse events.^11^,^13^

In fact, a recently published randomized cross-over control study compared the effect of a conventional 30-hour limit call system to two models employing a 16-hour limit (every fifth night overnight call, referred to as Q5; or night float, referred to as NF) on sleep duration, admission volumes, educational opportunities, number of handoffs and resident satisfaction.^7^ Both 16-hour systems increased the duration of sleep for interns while on call (NF) or post call (Q5), but this came at a cost of reducing continuity of care by increasing the number of handovers (from 3 to as many as 9) and the average number of interns caring for a given patient (from 3 to as many as 5).^7^ Since the vast majority of patient complaints to the provincial or state regulatory bodies are related to poor doctor-patient communication,^19^ these changes may further undermine the profession’s reputation and the public’s trust.

### Evaluating the literature

The effect of restricting resident duty hours on patient safety is unclear, with some reports suggesting improved mortality for both medical and surgical patients, improved quality of life, quality and safety of patient care, and resident education,^20^–^22^ while others fail to show any change in outcomes,^23^–^28^ and at least one showing increased concern among interns about making a serious medical error.^29^ Heterogeneity of outcomes in these studies is likely explained by the differences in research methodologies, with most studies using an observational pre-post design, thereby limiting extrapolation from observed associations to true cause and effect.^20^ These studies are also at risk of time-series bias. Some studies were underpowered to detect differences, or used self-reported measures that are prone to bias (e.g., quality of life). Lastly, meta-analyses and systematic reviews on this subject are likely compromised due to publication bias against negative studies, as well as by the heterogeneity in outcomes studied among different specialties.

### Economic consequences

The effect of such changes on a Canadian healthcare system that is already at capacity is unpredictable, but potential costs are projected to be quite high.^30^ In one Canadian post-graduate training centre, moving the call conversion time by just two hours to account for shortened shifts was projected to lead to over $600,000 per year in additional costs for a single institution alone. Such shifts, designated as “conversion calls”, are those shifts where a home call is converted to an in-house overnight call if work continues past a certain hour. Residents who convert their calls would thus be paid at the (slightly higher) in-house stipend rate. In this centre, conversion calls accounted for only 11% of all call shifts yet led to hundreds of thousands of dollars in unexpected costs. With such predictions likely to encompass the entire spectrum of call shifts (in-house, home and qualifying call), it comes as no surprise that total costs are expected to skyrocket. With cash-strapped governments and medical schools, the question of where such funds may be procured is a difficult one to answer.

Additionally, shorter working hours may necessitate an expansion in resident quotas, leading to greater expenses for health ministries and residency programs, or lower per-capita salaries for trainees. The need for greater resident coverage may lead to an artificial inflation of residency training spots, leading to “overproduction” of qualified residents for a limited number of staff positions, a mismatch that is already evident in some specialties.^31^

### Effect on staff physicians

An extension of this issue is a debate whether work-hour restrictions will eventually encompass staff physicians – either through professional standards of care or provincial legislation. The effects of such a restriction would require staff physicians in underserviced areas to make a difficult choice between declining urgent interventions to comply with the rules (and making patients suffer as a result) or proceeding with interventions and exposing themselves to personal and professional liability.

Lack of residents may also force subspecialty fellows (currently sheltered from many in-house calls) and staff physicians to cover general medicine and surgery calls, which may impair their quality of life and educational opportunities.^32^ Many staff physicians already work longer hours than their residents, sometimes unbeknownst to their trainees, in order to meet the increasing demands of teaching, running educational programs, doing administrative work, and conducting research in addition to clinical workload. Implementing limits on residents’ workloads without strategies to do the same for staff physicians may result in an unintended shift of work to the already overburdened staff physicians, with potential negative impact on the quality and safety of patient care, as well as quality of clinical teaching, educational programs, and research.^33^ The extreme of this extrapolation may see staff physicians forfeiting academic positions because of their unsustainability,^32^ and instead choosing to go with service-only assignments in the community, putting academic programs further at risk.

### Resident work-life balance

The greatest impact of the proposed change would be felt by residents. The reported improvement in quality of life with implementation of the 16-hour restriction may have been borne out by interns only, with senior residents reporting worsened quality of life, likely due to shift of work from junior to senior trainees.^11^ Since a large proportion of interns on medicine and surgery rotations are off-service and will never experience the negative effects of this work redistribution, medicine and surgery trainees have to shoulder the burden of worsened quality of life and increased work as they progress through their training, which may negatively influence overall morale, team dynamics, and even interest in these specialties as potential career options.

Losing the post-call day may negatively impact the quality of life of some residents,^29^,^34^ who view it as a chance to do errands and attend personal/family events. Night-float systems would require residents to leave their families on a nightly basis for the duration of that block, during which they will miss formalized teaching and other curricular day-time events.^35^ In one randomized trial, opportunities to attend daily rounds decreased by 25% when participants switched to a night-float call structure.^7^ In other studies, assessments of amount and quality of teaching declined as rated by attending physicians,^5^ while trainees noted a general deterioration in their satisfaction with educational activities.^33^ Although some programs have expanded curricula to allow for evening teaching sessions by attending physicians,^36^ the content delivered during these sessions may be limited by faculty availability – the same faculty who already face an increase in their clinical duties to compensate for work previously done by residents.^32^ Taken together, these changes may inadvertently lengthen the duration of residency in order to meet core educational requirements – a change that would cause uproar among medical students and residents who already endure training programs longer than any other occupation.

Residents may also lose their call stipend given that they are technically no longer on call. Since the call stipend increases resident salaries on medical/surgical services by as much as 14%, this would have a significant financial impact, especially for residents with families and dependants. Losing this income may actually worsen resident well-being in some of the most demanding programs by increasing their financial burden and stress levels.

### Moving forward on duty hours: a national consensus

Just because something is intuitive does not mean it is right. Proponents of duty hour reductions have often claimed that shortening work hours would, intuitively, lead to a concomitant decrease in medical errors and an apparent improvement in resident quality of life. Unfortunately, this has not been the case thus far, at least not on a consistent basis in the current literature. In a similar vein, those studies that have failed to show a reduction in medical errors often point to an increase in handovers as one potential cause. Those who advocate for duty-hour reductions state that improving handovers should lead to an increase in patient safety. Once again, intuitively this makes sense, but whether such an assumption holds true is as yet unknown and unfortunately has not been proven in the current body of research.

At the end of the day, there is a given amount of work that needs to be done in a given time. Introducing inflexible work-hour limits does not reduce the total amount of work to be done, it simply redistributes it. This concept of curtailing hours without a proportionate decrease in workload is known as work compression.^37^ We need to examine these various aspects and realize that sometimes the solution may be to keep things the same, at least when it comes to number of hours worked. What may be more important is targeting resident work type and intensity. For example, reducing unnecessary pages in the middle of the night would allow residents to work uninterrupted for longer periods and potentially complete work in a more efficient manner. Additionally, hiring allied health personnel such as nurse practitioners and physician assistants to help with the administrative side of medicine (such as completing long term care referrals or contacting family physicians) would help immensely. But perhaps paradoxically, attempting to bring about such changes to the culture of medicine may be more difficult than simply instituting rigid duty hour restrictions.

With all of this in mind, implementation of duty hours in Canada can potentially take two paths. The default is to follow in the footsteps of Quebec and mandate blanket duty hour restrictions for the whole country and across residency programs based on a single arbitration ruling and using limited evidence from mostly observational studies from the US. Alternatively, Canadians can assume a leadership role and develop call models that account for the complexity of this problem. We believe that the ‘one size fits all’ approach cannot be applied to resident work hours. As mentioned earlier, each specialty functions uniquely and it is in this context that we must move forward when examining call structures. We can study work patterns of practicing physicians in various settings (urban/rural/family/specialist) to determine variations and ensure that residency training takes that into account. We can conduct randomized trials to better assess various call models and duty hour patterns among different specialties (i.e., admitting services, such as medicine and general surgery, vs. non-admitting services, such as emergency medicine, radiology, and anesthesia). The Royal College of Physicians and Surgeons of Canada has already spearheaded such initiatives with a National Steering Committee hoping to examine these very issues.^38^ With a concerted national effort, it is our hope that any changes that are implemented are well researched and studied in a Canadian context with all stakeholders in mind, and that rushed, “patch-work” solutions to complex system-wide problems do not result in potentially detrimental outcomes.

## Conclusion

In summary, although there are certainly good intentions behind the proposed change to a shortened workday, we encourage all of the involved stakeholders to consider the potential unintended consequences outlined in this article and the impact they may have on all aspects of the healthcare system.

We also encourage residents to remember that medicine is more than a job - it is a calling. It requires personal sacrifice, but also offers rewards in so many ways. Our patients trust us with their lives, and our obligation is to always put that trust ahead of our personal interests. It is a privilege to learn the art alongside those who have mastered it before us. Indeed, the more time we spend with patients, the better physicians we become. Whatever changes are implemented, we hope that they do not result in physicians becoming time-card stamping bearers of medical knowledge who lose their connection with patients, and are unprepared and unwilling to serve them outside their duty hours.
